# Identification of Microbial Dark Matter in Antarctic Environments

**DOI:** 10.3389/fmicb.2018.03165

**Published:** 2018-12-19

**Authors:** Jeff S. Bowman

**Affiliations:** ^1^Scripps Institution of Oceanography, University of California, San Diego, La Jolla, CA, United States; ^2^Center for Microbiome Innovation, University of California, San Diego, La Jolla, CA, United States

**Keywords:** Antarctica, 16S rRNA, glacier, sea ice, cryoconite, sediment, permafrost, snow

## Abstract

Numerous studies have applied molecular techniques to understand the diversity, evolution, and ecological function of Antarctic bacteria and archaea. One common technique is sequencing of the 16S rRNA gene, which produces a nearly quantitative profile of community membership. However, the utility of this and similar approaches is limited by what is known about the evolution, physiology, and ecology of surveyed taxa. When representative genomes are available in public databases some of this information can be gleaned from genomic studies, and automated pipelines exist to carry out this task. Here the paprica metabolic inference pipeline was used to assess how well Antarctic microbial communities are represented by the available completed genomes. The NCBI’s Sequence Read Archive (SRA) was searched for Antarctic datasets that used one of the Illumina platforms to sequence the 16S rRNA gene. These data were quality controlled and denoised to identify unique reads, then analyzed with paprica to determine the degree of overlap with the closest phylogenetic neighbor with a completely sequenced genome. While some unique reads had perfect mapping to 16S rRNA genes from completed genomes, the mean percent overlap for all mapped reads was 86.6%. When samples were grouped by environment, some environments appeared more or less well represented by the available genomes. For the domain Bacteria, seawater was particularly poorly represented with a mean overlap of 80.2%, while for the domain Archaea glacial ice was particularly poorly represented with an overlap of only 48.0% for a single sample. These findings suggest that a considerable effort is needed to improve the representation of Antarctic microbes in genome sequence databases.

## Introduction

The Antarctic continent represents a complex mosaic of microbial habitats. At the continental margin are highly productive coastal seas which transition sharply to the oligotrophic Southern Ocean. Tidewater glaciers and ice shelves bridge the gap between the terrestrial and marine environments, while – along with terrestrial glaciers – providing unique microbial habitats in cryoconite holes, melt ponds, and subglacial lakes and streams. The complex topography of Antarctica provides for polar deserts and meltwater lakes varying in salinity from nearly fresh to near saturation. This environmental complexity and the isolation of the Antarctic from other continents has inspired over 100 years of microbiological research (McLean, 1918). However, only in the last few decades have DNA sequencing and other molecular methods allowed for the genetic and phylogenetic characterization of single celled members of the domains Eukarya, Bacteria, and Archaea.

Sequencing of the 16S rRNA gene has emerged as the *de facto* standard for determining the diversity of bacterial and archaeal communities. Although the maximum resolution of a diversity analysis by 16S rRNA gene sequencing is insufficient to identify many phylogenetically similar but genetically distinct strains, community structure derived from 16S rRNA gene sequencing does indicate the genetic structure of the community (Bowman and Ducklow, 2015). The efficacy of 16S rRNA gene sequencing studies has been improved in recent years by the stabilization of standard methods around the Illumina MiSeq sequencing platform, which provides high quality, high throughput sequencing of relatively short amplicons. Further aiding microbial diversity analysis are improved primers that broadly amplify across the domains Bacteria and Archaea (Walters et al., 2015), and new methods to denoise Illumina MiSeq data (Callahan et al., 2016; Amir et al., 2017). These methods allow microbial community structure to be resolved to the level of unique reads.

Despite the inaccessibility of much of the Antarctic continent, there have been numerous efforts to assess the taxonomic and genetic diversity of Antarctic microbial habitats. Scientific work following an initial assessment of microbial community structure in a given Antarctic environment may be limited, however, by the availability of completed genomes and model strains. These are necessary to fully understand the evolution, adaptation, and physiology of Antarctic microbes. Microbial clades that may be coarsely identified taxonomically, but for which little is known about their genetics, physiology, and ecological role, are considered “microbial dark matter” (Marcy et al., 2007) and are good targets for new studies and technological innovations.

To provide a status report on our understanding of Antarctic microbial diversity and the extent of microbial dark matter in different Antarctic environments, the available Illumina MiSeq studies were aggregated by environment, reanalyzed to the level of unique sequences, and a phylogenetic placement approach (Matsen et al., 2010) was applied to compare sequence identity to those closest completed genomes available in the public Genbank repository. The phylogenetic distance between environmental sequence reads and the closest completed genome provides an estimate of uncharacterized microbial diversity in these samples, and a novel view of the extent of microbial dark matter in Antarctic environments and the putative taxonomy of uncharacterized microbes.

## Materials and Methods

Datasets were identified on the NCBI SRA by search with the following syntax: Antarctica [All Fields] AND X metagenome [Organism], where X was an environment deemed relevant to Antarctica. These included “aquatic,” “freshwater,” “glacier,” “hypersaline lake,” “ice,” “lake water,” “marine,” “marine sediment,” “metagenome,” “microbial mat,” “rock,” “salt lake,” “seawater,” “sediment,” “soil,” “soil crust,” “snow,” and “terrestrial.” The goal of this search was not to carry out an exhaustive search for Antarctic datasets, but to capture nearly all the datasets available on SRA. To confirm the completeness of this search, an additional Google Scholar search was carried out using the search terms “Antarctica” and “Illumina.” The first 1,000 hits were reviewed for any studies that were not captured in the SRA search. Studies that used repositories other than SRA were not included.

Run tables were aggregated for all search results and filtered to include only amplicon studies that relied on the Illumina MiSeq or HiSeq platforms. Samples that were derived from host environments were also excluded from further analysis. Runs that were obviously the result of amplification of genes other than the 16S rRNA gene (e.g., 18S rRNA genes and intergenic transcribed spacer regions) were removed, as were studies where the data were clearly not demultiplexed when it was uploaded to the SRA. The remaining runs were downloaded using the fastq-dump command from the NCBI’s SRA Toolbox, with the – split-spots and – skip-technical flags. Here and elsewhere, Gnu Parallel was used to parallelize operations (Tange, 2011).

The consensus environment for each downloaded run was determined by evaluating the run metadata and, when available, any papers citing the run, study, or BioProject accession number. Because of ambiguity between freshwater lake sediments, hypersaline lake sediments, and marine sediments (e.g., samples associated with PRJNA387720), all sediments were classified as “sediment.”

Because not all of the data were derived from paired-end runs and many read pairs could not be merged, only the forward read was considered in this analysis. Quality control of the forward reads was carried out with the dada2 package (Callahan et al., 2016) in R (R Core Team, 2014). Reads were trimmed at the first position with a quality score below 20, reads with fewer than 75 bases were discarded, and the remaining reads denoised. Unique (non-redundant) reads were evaluated to determine their taxonomic domain using the CM scan function in Infernal (Nawrocki and Eddy, 2013) against covariance models for the domains Eukarya, Archaea, and Bacteria downloaded from the Rfam database (Nawrocki et al., 2015). Reads were assigned the domain for which they received the lowest *E*-value. Only reads identified as belonging to the archaea and bacteria were considered further.

The bacterial and archaeal reads associated with each run were analyzed using the paprica pipeline (Bowman and Ducklow, 2015). Paprica uses Infernal (Nawrocki and Eddy, 2013) and pplacer (Matsen et al., 2010) to place query reads on a phylogenetic tree constructed from full-length 16S rRNA genes extracted from completed genomes in Genbank. In this way, paprica makes a direct association between the query reads and the nearest phylogenetic neighbor with a completed genome. This association was used to make inferences about the degree of microbial dark matter present within different Antarctic environments. The paprica database used in this study was created on August 9, 2018, and thus includes completed Genomes that were available in Genbank on that date. The -unique flag was used for all scripts to enable tracking of unique reads through the paprica pipeline.

## Results

**FIGURE 1:**
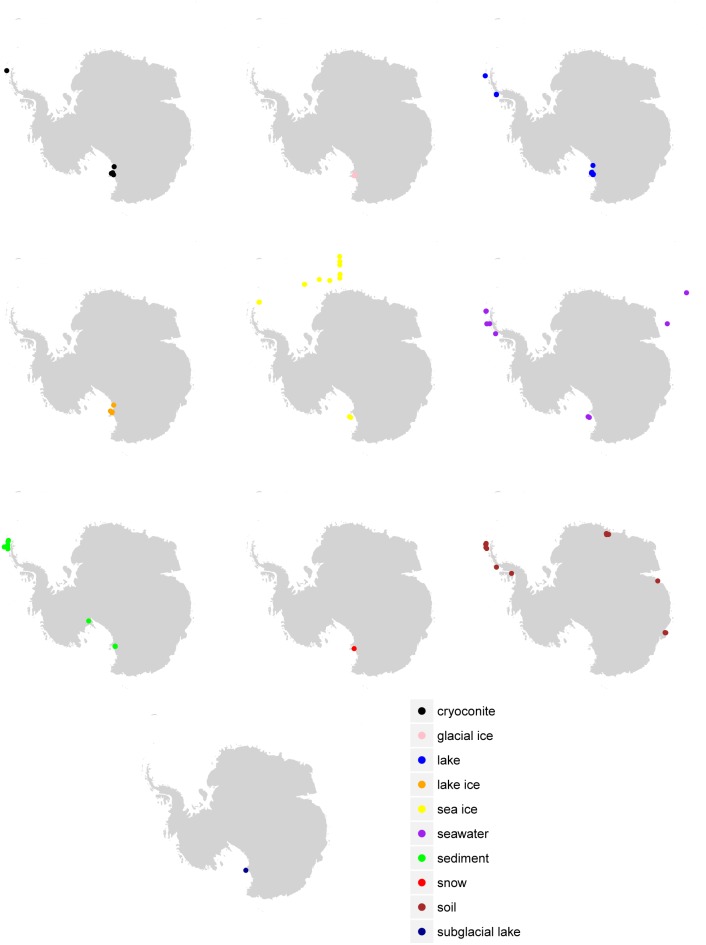
Sample location by environment. Sample locations (where available in the metadata) are given according to the final consensus environment.

This study identified 1,810 valid SRA runs that passed the selection criteria (Supplementary Table S1). These runs were associated with 39 BioProjects and consisted of 246,417,512 total forward reads. Of these runs, 1,772 had reads associated with the domains Bacteria and Archaea. 68,304,516 reads failed QC while an additional 13,063,077 reads could not be assigned to a domain at the specified cutoff. After QC and domain assignment the analyzed runs contained 163,272,230 reads (133,618,531 Bacteria and 29,653,699 Archaea). Consensus environments for runs, pulled from the run metadata and publications, included cryoconite (*n* = 168), glacial ice (*n* = 9), lake (*n* = 328), lake ice (*n* = 9), rock (*n* = 0), seawater (*n* = 221), sea ice (*n* = 69), sediment (*n* = 288), subglacial lake (*n* = 21), snow (*n* = 13), and soil (*n* = 684) (Figure 1 and Table 1). Samples associated with some BioProjects could not be used because of archival errors. These included PRJNA386567 (Frisia et al., 2017) which lacked quality data, and PRJNA480849 and PRJNA396917 (no citations available) which were not demultiplexed.

**Table 1:** Summary of BioProjects used in this study.

BioProject	Center name	Release date	Consensus environment	Citation
PRJEB11496	CENTRAL MICHIGAN UNIVERSITY	12/24/2015	Marine sediment	Learman et al., 2016
PRJEB11497	CENTRAL MICHIGAN UNIVERSITY	12/24/2015	Marine sediment	Learman et al., 2016
PRJEB11689	UNIVERSITY OF WARWICK	12/1/2017	Soil	
PRJEB14880	UNIVERSITY OF CALIFORNIA SAN DIEGO MICROBIOME INIT	7/23/2016	Marine sediment	Beaupré and O’Dwyer, 2017
PRJEB20869	UNIVERSITY OF CALIFORNIA SAN DIEGO MICROBIOME INIT	8/25/2017	Lake	
PRJEB21441	UNIVERSITY OF CALIFORNIA SAN DIEGO MICROBIOME INIT	8/25/2017	Soil	
PRJEB22851	EUROPEAN MOLECULAR BIOLOGY LABORATORY	12/2/2017	Lake	Kleinteich et al., 2017
PRJEB23732	UNIVERSITY OF CAMBRDGE	12/20/2017	Soil	
PRJEB25155	UNIVERSITY OF NEUCHATEL	7/3/2018	Lake	
PRJNA244335	LOUISIANA STATE UNIVERSITY	7/31/2014	Subglacial lake	Christner et al., 2014
PRJNA254078		6/15/2015	Seawater	
PRJNA278982	LOUISIANA STATE UNIVERSITY	7/23/2015	Subglacial lake	Vick-Majors et al., 2016
PRJNA280421	UNIVERSIDAD MAYOR	4/18/2015	Seawater	Moreno-Pino et al., 2016
PRJNA282540	LOUISIANA STATE UNIVERSITY	4/28/2016	Glacial ice	
PRJNA296701		10/8/2015	Cryoconite	Webster-Brown et al., 2015
PRJNA304081		6/30/2016	Soil	Tahon et al., 2016
PRJNA305344		12/13/2015	Soil	Tytgat et al., 2016
PRJNA305852		12/19/2015	Lake	de Scally et al., 2016
PRJNA306790		1/11/2016	Sea ice	Bowman and Deming, 2016
PRJNA315812		4/4/2016	Seawater	Rozema et al., 2017
PRJNA317932	CSIRO	4/27/2017	Soil	Bissett et al., 2017
PRJNA320505	INSTITUTE OF BIOCHEMISTRY AND BIOPHYSICS POLISH A	5/10/2016	Cryoconite	
PRJNA344476		10/13/2016	Seawater	Bowman et al., 2017
PRJNA355879		12/5/2017	Sea ice	Eronen-rasimus et al., 2017
PRJNA357685		12/24/2016	Soil	Yan et al., 2017
PRJNA359740		1/8/2017	Soil	
PRJNA386506		5/12/2017	Marine sediment	Bendia et al., 2018
PRJNA387720		10/6/2017	Marine sediment	
PRJNA395496		7/24/2017	Soil	
PRJNA398047		10/4/2017	Seawater	
PRJNA401502		9/6/2017	Cryoconite	
PRJNA401941		9/7/2017	Cryoconite	Sommers et al., 2018
PRJNA415906		2/18/2018	Soil	Rippin et al., 2018
PRJNA433184		2/6/2018	Soil	Zhang et al., 2018
PRJNA433310		2/7/2018	Soil	Zhang et al., 2018
PRJNA433331		2/7/2018	Soil	Zhang et al., 2018
PRJNA433699		2/9/2018	Marine sediment	
PRJNA471123		5/12/2018	Soil	

**FIGURE 2:**
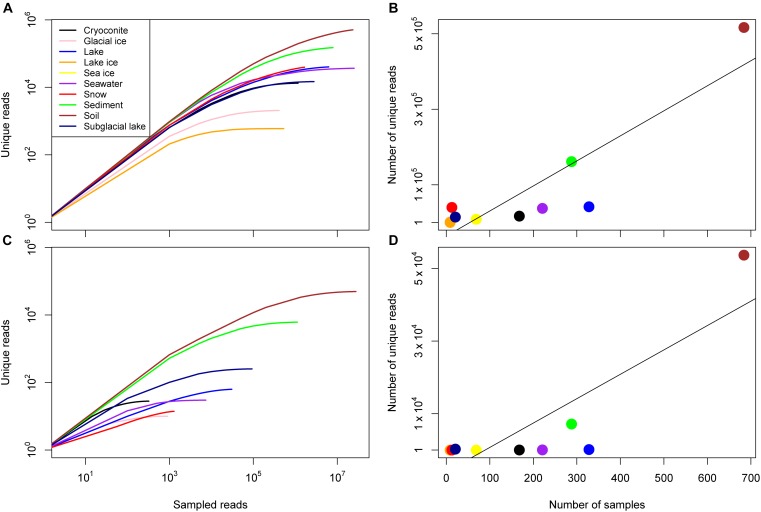
Sample diversity for the domain Bacteria and Archaea. **(A)** Rarefaction curves for all consensus environments for bacteria given on a log-log scale. **(B)** The number of unique reads identified in each consensus environment as a function of the number of samples, the line of best fit reflects a linear relationship (*R*^2^ = 0.78, *p* = 2 × 10^-4^). **(C)** Rarefaction curves for all consensus environments for archaea given on a log–log scale. Note that no archaea were identified in lake ice or sea ice samples. **(D)** The number of unique reads identified in each consensus environment as a function of the number of samples, the line of best fit reflects a linear relationship (*R*^2^ = 0.69, *p* = 9 × 10^-4^).

The number of unique reads varied widely between environment and was strongly correlated with the number of samples for archaea and bacteria (Figure 2 and Table 2). For bacteria, the number of samples associated with a given environment accounted for 77.0% of the variance in the number of unique sequences across environments (Pearson correlation, *p* = 0.0002). Some environments, however, had a higher number of unique reads than anticipated by this model. These included environments with very few samples including snow, subglacial lake, lake ice, and soil. For archaea, the number of samples accounted for 69.4% of the variance the number of unique reads (Pearson correlation, *p* = 0.0017). Similar to bacteria, snow, subglacial lake, lake, and soil had a higher number of unique reads than predicted by sample number.

**Table 2:** Read data for each environment.

	Cryoconite	Glacial ice	Lake	Lake ice	Sea ice	Seawater	Sediment	Snow	Soil	Subglacial lake
Number of samples	168	9	287	9	69	218	270	13	508	21
Number of final reads × 10^6^	2.837392	1.403345	13.164907	1.151896	0.594581	47.326081	16.22292	4.721608	70.63853	5.210974
Number of final reads, Bacteria × 10^6^	2.836965	1.40178	13.128534	1.151896	0.594535	47.317886	15.04291	4.720207	42.30966	5.114157
Number of final reads, Archaea × 10^6^	0.000427	0.001565	0.036373	0	0.000046	0.008195	1.180008	0.001401	28.32887	0.096817
Number of normalized reads, Bacteria × 10^6^	1.170712	0.409695	6.27037	0.52973	0.376019	25.230212	7.973523	1.67086	23.810962	2.789416
Number of normalized reads, Archaea × 10^6^	0.00035	0.000917	0.030706	0	0.000038	0.007419	1.117945	0.0013	27.794788	0.093139
Number of unique reads, Bacteria	133351	2073	40711	598	7711	36955	152348	40405	514020	14826
Number of unique reads, Archaea	28	10	63	NA	2	30	6154	14	49669	254
Mean map ratio, Bacteria	0.88	0.91	0.86	0.91	0.87	0.80	0.90	0.86	0.87	0.90
Mean map ratio, Archaea	NA	0.48	0.93	NA	NA	0.86	0.83	0.50	0.83	0.85

**FIGURE 3:**
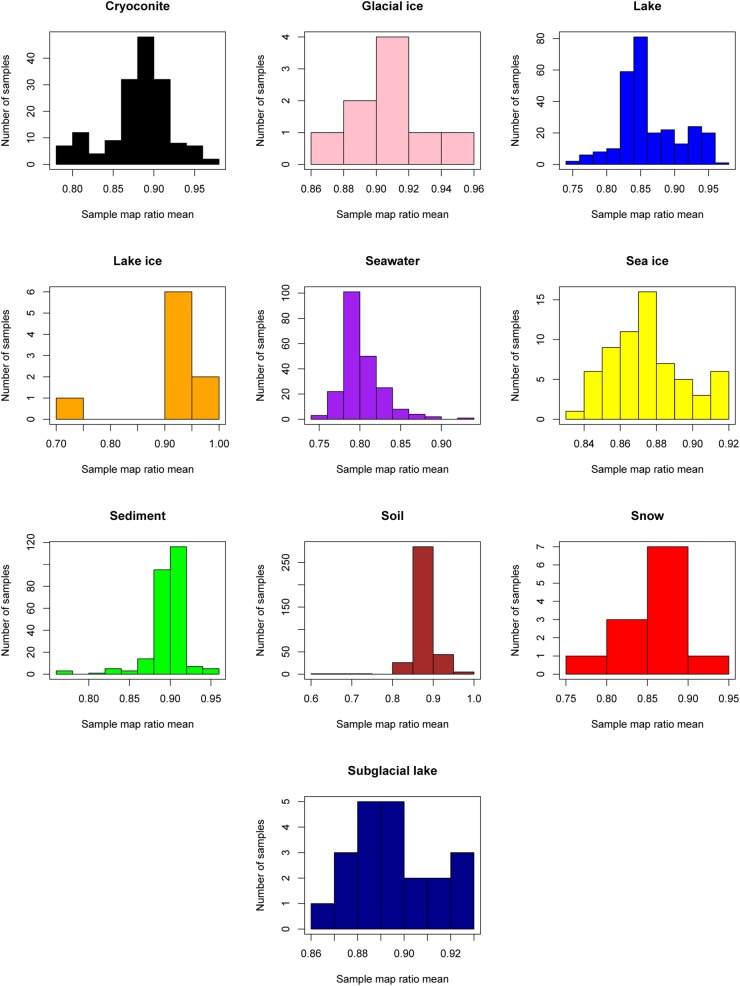
Sample mean map ratios for the domain Bacteria. For each consensus environment the distribution of mean map ratios is given. Only samples with greater than 1,000 reads assigned to the domain Bacteria are shown in the distribution.

**FIGURE 4:**
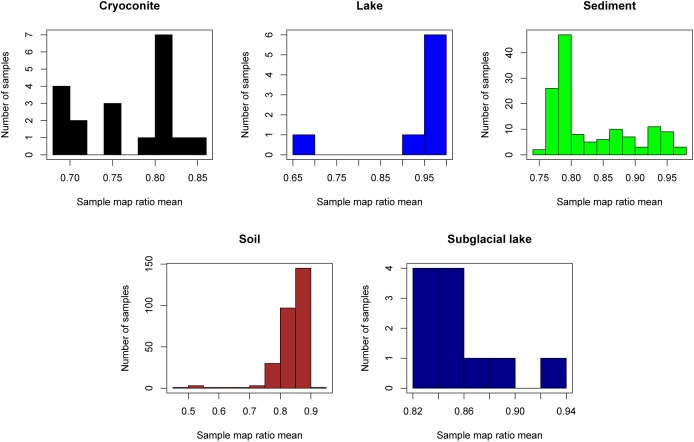
Sample mean map ratios for the domain Archaea. For each consensus environment the distribution of mean map ratios is given. Only samples with greater than 1,000 reads assigned to the domain Archaea are shown in the distribution. Due to the small number of samples with sufficient archaeal reads for glacier ice (*n* = 1), lake ice (*n* = 0), snow (*n* = 1), sea ice (*n* = 0), and seawater (*n* = 3), these environments are not shown.

The “map ratio” variable, calculated by pplacer (Matsen et al., 2010) as the percent identity between the query read and reference sequence, was used as the primary indicator of how well 16S rRNA gene reads were represented by completed genomes in Genbank (i.e., the paprica database). The mean map ratio distribution by sample, limited to samples with more than 1,000 reads assigned to the bacteria or archaea, was used to identify environments that may have microbial communities more poorly represented by completed genomes of bacteria (Figure 3) and archaea (Figure 4). Mean map ratios ranged from 0.60 (SRR3455314, soil) to 0.97 (SRR6008356, cryoconite) for bacteria, and from 0.48 (SRR2006327, glacial ice) to 0.97 (SRR5535794, sediment) for archaea. Samples with more than 1,000 reads but a mean map ratio below 0.8 were flagged for further investigation. For bacteria these included samples from cryoconite (*n* = 7), lake (*n* = 16), lake ice (*n* = 1), seawater (*n* = 126), snow (*n* = 1), sediment (*n* = 3), and soil (*n* = 3) environments. For the archaea these included samples from glacial ice (*n* = 1), lake (*n* = 15), snow (*n* = 1), sediment (*n* = 75), and soil (*n* = 40).

**FIGURE 5:**
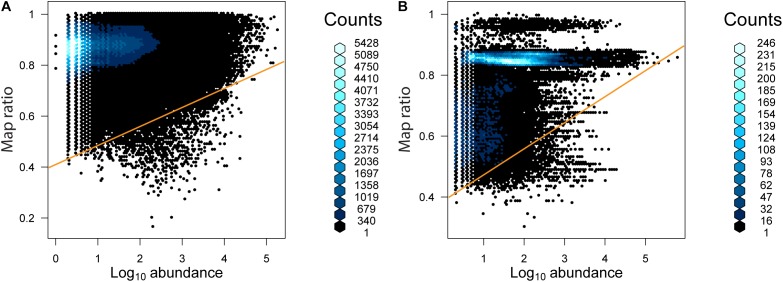
The abundance of unique reads as a function of map ratio for **(A)** bacteria and **(B)** archaea. The abundance of unique reads was determined within each consensus environment (i.e., each unique read may be tallied more than once in different consensus environments). The distribution of data is displayed via a hexagonal density plot, with the color of the hexagons representing the density of the data.

The relationship between the abundance of unique reads (across the entire dataset) and map ratio (Figure 5 and Table 3) was used to identify unique reads that were abundant in individual samples but poorly represented by completed genomes. Unique reads for which the map ratio was less than 0.075 × read abundance + 0.4 were flagged for further inspection. These parameters were selected arbitrarily to objectively subset a manageable number of reads. For bacteria 1,675 unique reads met this criterion, while 1,949 unique reads met this criterion for archaea. The 10 most abundant phylogenetic edges (branches or tips) within any environment associated with these unique reads were evaluated further to determine which sequenced genomes represent groups with considerable uncharacterized diversity (Table 3). The most abundant low map ratio edge for domain Bacteria accounted for 3.9% total seawater reads and placed with *Sulfitobacter pseudonitzschia* SMR1, represented by Genbank genome GCF002222635.1. Classification of a representative sequence using the Ribosomal Database Project (RDP) classifier (Wang et al., 2007) identified the read as belonging to the phylum Proteobacteria. The most abundant low map ratio edge for domain Archaea accounted for 1.2% of total soil reads and belonged to the family *Haloarculaceae*. RDP classified a representative read as domain Archaea.

**Table 3:** Abundant phylogenetic edges with low map ratio values.

Edge	Unique reads	Abundance	Mean map ratio	Taxon^1^	RDP (50%)^2^	Predominant environment
**Bacteria**						
222	166	979594	0.663945247	GCF002222635.1 *Sulfitobacter pseudonitzschiae* SMR1	Phylum Proteobacteria	Seawater
10015	24	111525	0.517836875	FCB Group	Class Cyanobacteria	Soil, seawater
475	36	104435	0.629734278	Brucellaceae	Phylum Proteobacteria	Seawater
696	40	99713	0.5506075	GCF000183665.1 Candidatus *Liberibacter solanacearum* CLso-ZC1 CLso-ZC1	Domain Bacteria	Seawater
9917	2	94871	0.746377	*Oscillatoriophycideae*	Chloroplast (genus *Bacillariophyta*)	Seawater, sediment
219	11	68825	0.669268636	GCF002158905.1 *Yoonia vestfoldensis* SMR4r	Class Alphaproteobacteria	Seawater
53	23	67685	0.632538435	GCF000296215.2 *Bradyrhizobium* sp. CCGE-LA001 CCGE-LA001	Domain Bacteria	Seawater
960	81	57654	0.581226012	GCF000815025.1 *Coxiella* endosymbiont of *Amblyomma americanum*	Root	Snow
7058	11	50260	0.645450273	GCF001399775.1 *Thermus aquaticus* Y51MC23 Y51MC23	Domain Bacteria	Seawater
875	20	39762	0.599264	Rhodospirillum	Domain Bacteria	Seawater
**Archaea**						
107	224	329322	0.488955781	*Haloarculaceae*	Domain Archaea	Soil
341	83	83108	0.556996494	GCF002214165.1 Candidatus *Microarchaeota archaeon* Mia14	Root	Soil, sediment
127	48	76379	0.502742583	GCF900079125.1 *Methanoculleus bourgensis*	Domain Archaea	Soil
347	44	69891	0.491039114	Candidatus *Nitrosopumilus sediminis* AR2	Genus *Nitrososphaera*	Sediment, soil
340	7	53978	0.679012	Archaea	Root	Soil
431	50	50964	0.51836778	GCF000317795.1 *Caldisphaera lagunensis* DSM 15908 DSM 15908	Domain Archaea	Soil
225	26	43723	0.463009192	Euryarchaeota	Domain Archaea	Soil
126	14	35384	0.511628	GCF000304355.2 *Methanoculleus bourgensis* MS2	Domain Archaea	Soil
348	28	17068	0.504448786	GCF002156965.1 Candidatus *Nitrosomarinus catalina* SPOT01	Genus Nitrososphaera	Soil
364	31	15033	0.463741968	GCF000018305.1 *Caldivirga maquilingensis* IC-167	Domain Archaea	Soil

*Abundance values include only those reads with low map ratios, not all reads placed to the indicated edge. ^*1*^Taxonomy of the phylogenetic edge on the paprica reference tree. ^*2*^Classification of a representative read for that edge using the Ribosomal Database Project classifier (Wang et al., 2007) with a 50% cutoff*.

As seen in the distribution of abundant unique reads with low map ratios (Table 3), the uncharacterized diversity for the bacteria was most pronounced in seawater. This is interesting given that the mean map ratio for seawater samples was not particularly low; individual samples with lower values were found in lake and soil environments (Figure 3). In seawater low map ratios were balanced by abundant, well characterized taxa with high map ratios, particularly *Pseudoalteromonas spongiae* UST010723-006 (map ratio = 0.98), Candidatus *Pelagibacter ubique* HTCC1062 (map ratio = 0.96), and *Alteromonas stellipolaris* LMG21856 (map ratio = 0.97). A precise classification of the low map ratio reads was not possible – as is expected for microbial dark matter – and in most cases the RDP classifier could not provide a classification below the level of domain. *S. pseudonitzschia* SMR1, the edge in seawater samples that had the lowest mean map ratio, was relatively abundant and correctly classified by RDP as belonging to the Proteobacteria.

Although lake ice and soil environments generally had high mean map ratios for bacteria, some samples from these environments were unusually low (Figure 3). For lake ice, sample ERR2204499 from BioProject PRJEB22851 was a clear outlier with a mean map ratio of 0.71. Although this sample had enough reads associated with the domain Bacteria to be considered for the mean map ratio analysis (*n* = 2,225), the sample was of very low diversity with only seven unique reads identified. All of these unique reads had fairly high map ratios except for one abundant read that placed with *Halomonas chromatireducens* AGD 8-3. The soil sample with the lowest map ratio (SRR3455314, BioProject PRJNA317932) also had relatively few reads (*n* = 1,207) but was comparatively diverse, with 59 unique reads. The most abundant among these belonged to an unclassified Betaproteobacteria with a map ratio of 0.62, *Verrucomicrobia* with a map ratio of 0.52, and *Blattabacterium punctulatus* CPUpc with a map ratio of 0.60. Verrucomicrobia and Betaproteobacetria are common in soil environments, and *Blattabacterium* spp. are obligate endosymbionts (Gil et al., 2004), thus reads placed to *B. punctulatus* CPUpc may have been associated with nemotades, tardigrades, or other metazoans common to Antarctic soils.

The sample with the lowest mean map ratio (0.48) for domain Archaea belonged to glacial ice (SRR2006327, BioProject PRJNA282540). Abundant unique reads in this sample with low map ratios mapped to *Haloarcula marismortui* ATCC 43049 represented by genome GCF_000011085.1 and *Ferroplasma acidiphilum* Y represented by genome GCF_002078355.1. Although the mean map ratios for soil samples were generally higher for domain Archaea, some soil samples had exceptionally low values. The lowest mean map ratio for soil was sample ERR2012973 (BioProject PRJEB21441) at 0.49. Abundant unique edges with low map ratios in this sample included *Metallosphaera curpina* Ar-4 (map ratio = 0.49) represented by genome GCF_000204925.1 and *Methanospirillum hungatei* JF-1 represented by genome GCF_000013445.1 (map ratio = 0.43).

## Discussion

The domains Bacteria and Archaea showed surprising differences in their relative abundance and in the number of unique sequences identified. Overall bacteria were better sampled than archaea, though this does not necessarily reflect any greater ecological importance in many environments. Only recently have primers been designed to broadly amplify across both domains (Walters et al., 2015), prior to this many studies focused on the domain Bacteria as a matter of expediency. Thus while no archaeal reads were identified in lake ice and sea ice, and very few in glacial ice and snow, this does not mean that archaea were absent from those physical samples. Archaea were comparatively well sampled in sediment and soil – environments that are known to host a considerable number of archaea – but the number of unique reads associated with the archaea in these environments was nearly an order of magnitude less than the number associated with the domain Bacteria, despite a similar number of sampled reads. This may reflect an overall lower phylogenetic diversity among the archaea or an analysis artifact, with the available primers and covariance models insufficient to capture the true archaeal diversity. The lack of archaeal sequence data was particularly pronounced for seawater, where archaea are known to play a considerable role in the marine nitrogen cycle and in dark carbon fixation (Tolar et al., 2016).

A key distinction between the Bacteria and Archaea in this analysis was the impact of read normalization on read abundance. The paprica pipeline normalizes read abundance by dividing the number of reads placed to an edge on the phylogenetic reference tree by the anticipated 16S rRNA gene copy number for that position on the tree. Because many bacteria and archaea have multiple copies of the 16S rRNA gene, this can have a major impact on the estimated abundance of these clades. Across the soil samples, for example, 42.3 × 10^6^ reads were associated with the domain Bacteria and 28.3 × 10^6^ with Archaea. After normalization only 23.8 × 10^6^ reads were associated with Bacteria (a 44% reduction), while 27.8 × 10^6^ were associated with Archaea (a 2% reduction; Table 1). Extrapolating these ratios to a hypothetical single sample suggests that the abundance of bacteria relative to archaea would be overestimated by a factor of nearly 2 if the data were not normalized.

While lake and glacial ice had the highest mean map ratios for bacteria, and lake the highest for archaea, no Antarctic environment was well represented by the available completed genomes in Genbank. All of the investigated environments had some samples with comparatively low mean map ratios, and all samples had some number of unique reads with low map ratios. The abundance of a *S. pseudonitzschia* SMR1 phylotype with a low map ratio in seawater samples indicates that even relatively well-sampled (ranked 4th out of 10 for number of samples per environment) environments contain considerable uncharacterized diversity. *S. pseudonitzschia* was isolated from the marine diatom *Pseudonitzschia* multi-series (Hong et al., 2015) suggesting that phytoplankton blooms – comparatively well-studied environments – may host their own microbial dark matter. It is important to note the difference between phylogenetic dissimilarity and sequence identity for such well-characterized taxa as *Sulfitobacter*; an uncharacterized strain may be most closely related to (e.g.) sequenced *Sulfitobacter* but nonetheless share little sequence identity. Overall the dissimilarity between environmental sequence reads and 16S rRNA genes from completed genomes is not surprising given the paucity of completely sequenced genomes from Antarctica. Because data on isolation environment is not typically included with genome metadata it is difficult to determine how many complete genomes of Antarctic bacteria and archaea have been sequenced. However, Bowman (2017) recently identified only 32 completely sequenced psychrophile genomes, suggesting that bacteria and archaea from the perennially cold Antarctic are not well represented.

A great number of valuable studies were excluded from this analysis based on technical limitations, including the use of older sequencing technologies such as Roche 454, or poor sequence quality. The rate of technological innovation for high-throughput sequencing methods has been extreme since the first 454 sequencing study in 2006 (Sogin et al., 2006), and the current primers and Illumina MiSeq methodologies reflect a maturation of this technology (e.g., Thompson et al., 2017). Although widely adopted these methodologies are not ubiquitous, however, and individual investigators must strive to adopt best practices for microbial ecology studies. Methodological errors are compounded by archival errors; several studies of interest could not be used because the data were not correctly uploaded to SRA. The most common error made was not demultiplexing at the time of submission; without a map file identifying barcodes and sample-specific metadata these data are meaningless to the wider community. Journals and funding agencies should continue to require that sequence data and appropriate metadata be archived at the time of manuscript submission or at the completion of a project; however, the current checks are insufficient to insure that data is discoverable and reusable.

Despite the vast size of the Antarctic continent, sampling for most environments was concentrated in just a few areas (Figure 1). The western Antarctic Peninsula and McMurdo regions were the most heavily sampled and accounted for nearly all terrestrial samples except for soil. Soil was sampled in several other locations in eastern Antarctica, namely Prydz Bay and in the Sor Røndane Mountains. Sea ice was sampled exclusively in the Ross Sea region (including McMurdo Sound) and the Weddell Sea. How much microbial diversity remains undiscovered because of this bias is a difficult question to answer. Certainly within these more densely sampled sites there are habitats in space and time that are undersampled, or that have not been sampled at all. The implications of this is clear from the relationships in Figure 3; while individual samples within environments may be sampled to saturation, this does not necessarily mean that the total diversity of the environment is well sampled. Future investigations will need to continue to focus on better understanding the environmental drivers of diversity within more heavily sampled regions, while expanding to include new areas that have not been included in previous sampling efforts.

## Data Availability Statement

All data used in this study are available from the NCBI SRA at the BioProjects listed in Table 1. Additional information on each included sample is provided in a table in the Supplementary Information.

## Author Contributions

JB conceived the study, carried out the analysis, and wrote the manuscript.

## Conflict of Interest Statement

The author declares that the research was conducted in the absence of any commercial or financial relationships that could be construed as a potential conflict of interest.
